# Synovial fluid hyaluronan mediates MSC attachment to cartilage, a potential novel mechanism contributing to cartilage repair in osteoarthritis using knee joint distraction

**DOI:** 10.1136/annrheumdis-2014-206847

**Published:** 2015-05-06

**Authors:** Thomas G Baboolal, Simon C Mastbergen, Elena Jones, Stuart J Calder, Floris P J G Lafeber, Dennis McGonagle

**Affiliations:** 1 Faculty of Medicine, Leeds Institute of Rheumatic and Musculoskeletal Medicine, University of Leeds, Leeds, West Yorkshire, UK; 2 Rheumatology & Clinical Immunology, University Medical Center Utrecht, Utrecht, The Netherlands; 3 NIHR-Leeds Musculoskeletal and Biomedical Research Unit, Chapel Allerton, Leeds Teaching Hospital Trust, Leeds, West Yorkshire, UK; 4 Department of Trauma and Orthopaedics, Chapel Allerton, Leeds Teaching Hospital Trust, Leeds, West Yorkshire, UK

**Keywords:** Knee Osteoarthritis, Osteoarthritis, Synovial fluid, Orthopedic Surgery

## Abstract

**Objectives:**

Knee joint distraction (KJD) is a novel, but poorly understood, treatment for osteoarthritis (OA) associated with remarkable ‘spontaneous’ cartilage repair in which resident synovial fluid (SF) multipotential mesenchymal stromal cells (MSCs) may play a role. We hypothesised that SF hyaluronic acid (HA) inhibited the initial interaction between MSCs and cartilage, a key first step to integration, and postulate that KJD environment favoured MSC/cartilage interactions.

**Methods:**

Attachment of dual-labelled SF-MSCs were assessed in a novel in vitro human cartilage model using OA and rheumatoid arthritic (RA) SF. SF was digested with hyaluronidase (hyase) and its effect on adhesion was observed using confocal microscopy. MRI and microscopy were used to image autologous dual-labelled MSCs in an in vivo canine model of KJD. SF-HA was investigated using gel electrophoresis and densitometry.

**Results:**

Osteoarthritic-synovial fluid (OA-SF) and purified high molecular weight (MW) HA inhibited SF-MSC adhesion to plastic, while hyase treatment of OA-SF but not RA-SF significantly increased MSC adhesion to cartilage (3.7-fold, p<0.05) These differences were linked to the SF mediated HA-coat which was larger in OA-SF than in RA-SF. OA-SF contained >9 MDa HA and this correlated with increases in adhesion (r=0.880). In the canine KJD model, MSC adhesion to cartilage was evident and also dependent on HA MW.

**Conclusions:**

These findings highlight an unappreciated role of SF-HA on MSC interactions and provide proof of concept that endogenous SF-MSCs are capable of adhering to cartilage in a favourable biochemical and biomechanical environment in OA distracted joints, offering novel one-stage strategies towards joint repair.

## Introduction

End stage osteoarthritis (OA) is inevitably associated with severe articular cartilage loss and joint failure.^1^ Joint replacement is the gold standard treatment but autologous chondrocyte implantation (ACI) may be used to treat isolated chondral lesions, which may be forerunners of OA.^2^ ^3^ However, ACI is limited to younger subjects (<40 years) and has shown some promise in this patient group,^4^ ^5^ but is not suitable for the majority of patients with OA. Surgical off-loading using realignment osteotomy is also associated with cartilage repair,^6^ ^7^ indicating an endogenous repair mechanism. For more advanced OA, spontaneous cartilage repair was considered impossible, despite aberrant remodelling elsewhere in the joint (including chondro-osteophyte formation).^8^ Recently, remarkable spontaneous cartilage repair in advanced OA has been shown following knee joint distraction (KJD) within as little as 8 weeks.^9^ ^10^

There is an emerging interest in the use of multipotential mesenchymal stromal cells, also termed mesenchymal stem cells (MSCs) and/or scaffolds for joint repair.^11–14^ The biological basis for KJD-associated spontaneous joint repair without addition of scaffolds, growth factors or exogenous cells is not understood, but clearly indicates a resident endogenous repair capacity. Joint resident MSCs and the local biochemical and biomechanical environment are anticipated to be central to this phenomenon.

We and others previously described a synovial fluid (SF) resident MSC population in OA, rheumatoid arthritic (RA) and non-arthritic joints, where elevated numbers were seen in early and advanced OA^15^ ^16^ and following meniscal injury.^17^ Thereafter, SF-MSCs were shown to be capable of participating in ligament regeneration.^18^ ^19^ Furthermore, studies in pigs have shown cartilage regeneration upon introduction of culture expanded MSCs into the synovial joint space and considerable repair in the sham control group, suggesting repair activity by resident MSCs.^20^ Recruitment of endogenous cells has also been shown to repair whole articular surfaces in rabbits.^14^

Key endogenous factors leading to intrinsic cartilage repair are likely associated with SF-MSCs, joint biomechanics and SF homeostasis including growth factors and hyaluronic acid (HA) composition. Given that SF-MSCs highly express CD44,^15^ ^18^ ^19^ ^21^ we hypothesised that interactions between CD44 and HA lead to the formation of a pericellular coat (HA-coat).^22–25^ These interactions in an OA environment might profoundly influence and potentially block MSC adhesion to cartilage. We further hypothesised that KJD might also affect these interactions. Herein, we show a critical molecular weight (MW) dependent role for SF-HA in determining SF-MSC interactions with cartilage in vitro and in vivo that opens up a hitherto unappreciated mechanism for understanding how resident SF-MSCs could be manipulated to develop better one-stage therapies for OA in KJD and other settings.

## Materials and methods

### Collection of human cartilage, SF and SF-MSCs

All samples were collected following written informed consent with relevant ethical approval. Cartilage samples were obtained during total knee replacement surgery. OA-SF and RA-SF was collected from patients undergoing joint replacement or aspirated during routine clinics. For SF samples, cells were pelleted at 16 000 rcf for 5 min and SF frozen at −80°C. SF-MSCs were derived and expanded as previously described.^16^

### Cartilage adhesion assay

SF-MSCs were dual-labelled with fluorescent micro-sized particles of iron oxide (FMPIO, Bang Laboratories). Macroscopically normal osteochondral (OC) plugs (8 mm diameter) were placed into a preformed 8 mm diameter well of sterile 2% agarose. FMPIO-SF-MSCs (p2-4, 5×10^4^ per OC plug) were resuspended in either a culture medium, OA-SF or RA-SF with or without addition of hyaluronidase (hyase, see online supplementary information) before being added to the OC plug and incubating overnight at 37°C. Thereafter, the cartilage surface was gently washed and adherent SF-MSCs were fixed in 3.7% formalin. Confocal microscopy was used to image attached cells.

### Animals

Mixed breed dogs were obtained from Utrecht University animal laboratory. The Utrecht University Committee of Experiments on Animals approved the study according to Dutch law (DEC: 2011.III.11.116).

### Knee joint distraction in Canine Groove model and injection of labelled MSCs

Canine Groove model of OA was bilaterally induced in the right and left stifle joints in three dogs.^26^ ^27^ After 10 weeks, KJD was performed on the right stifle joint for 5 days. The external fixation frame was placed onto the femur and the tibia under general anesthesia and pain medication. Autologous FMPIO-labelled MSCs (passage (p) 2) were recovered from frozen. Each knee received 5.6–8.7×10^6^ autologous cells injected into the synovial cavity in 1 mL saline supplemented with 5% canine serum 72 h after placement of the external fixation frames. Each animal was allowed to continue normal daily activities before euthanising 48 h after MSC injection, whereby the joints were dissected and fixed in formalin.

### Statistical analysis

Statistics were performed using SPSS Statistics V.21 (IBM, Portsmouth). Unless otherwise stated, all data were treated as non-parametric. Where applicable, paired analysis was done using the Wilcoxon Signed Ranks and non-paired using the Mann-Whitney U test. Correlations were calculated using the Spearman's rank correlation coefficient for non-parametric data. For further details please see online supplementary information.

## Results

### OA-SF is antiadhesive and limits cell attachment

**Figure 1:**
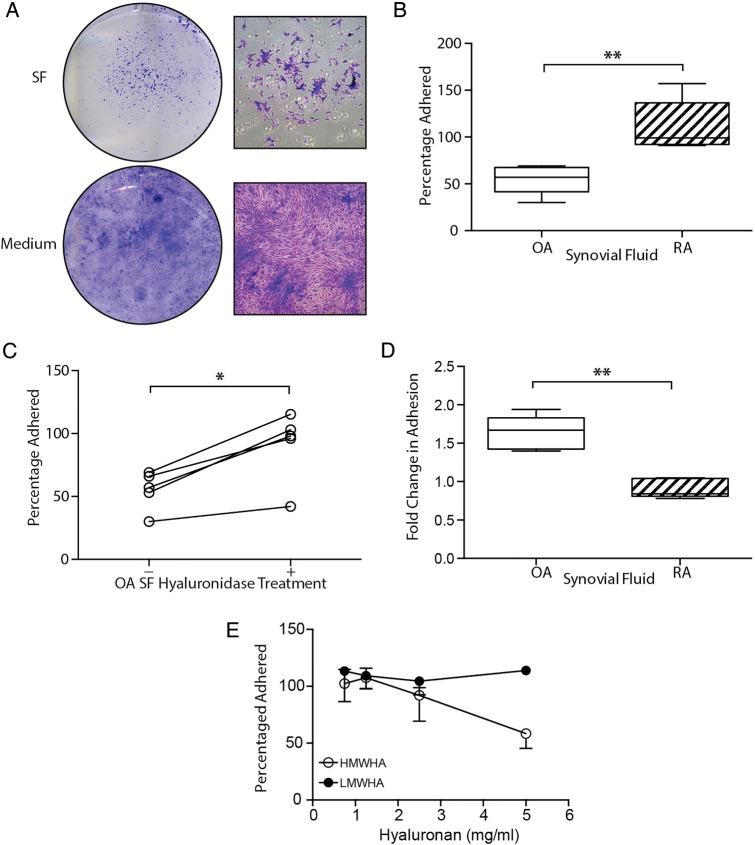
Antiadhesive nature of osteoarthritic (OA) synovial fluid (SF) on tissue culture plastic. (A) Direct plating of SF or replacement of SF by culture medium illustrating the extent to which synovial fluid-mesenchymal stromal cell (SF-MSC) adhesion is affected by osteoarthritic-synovial fluid (OA-SF). Gross (left) and magnified (right) images of culture morphology. (B) Differential adhesion of expanded SF-MSCs in OA-SF and rheumatoid arthritic-synovial fluid (RA-SF) (n=5 each, non-paired analysis). (C) Increase in adhesion of SF-MSCs in OA-SF after predigestion with hyase (n=5, paired analysis). (D) Differential effect of hyase treatment on SF-MSC adhesion in RA-SF and OA-SF (n=5 each, non-paired analysis). (E) Increasing concentrations of high, but not low molecular weight hyaluronan (HMWHA and LMWHA, respectively) inhibit the adhesion of SF-MSCs from three donors. *p<0.05, **p<0.001; individual samples are given in (C), median values with 25% (box) and 75% (whiskers) CI are presented in (B) and (D).

Recovery and expansion of SF-MSCs directly from SF was poor; however, by replacing SF with culture medium, cells readily adhered and proliferated on tissue culture plastic (figure 1A). To investigate to what extent SF inhibited MSC adhesion, we obtained RA-SF (n=5) and OA-SF (n=5) and determined the relative adhesion of culture expanded (p2-4) SF-MSCs in the presence of RA-SF and OA-SF. Adhesion of SF-MSCs in RA-SF was greater than that in OA-SF, with a mean twofold increase (p=0.008) in adhesion (figure 1B).

### Hyaluronidase rescues and HA potentiates MSC adhesion to plastic

HA is known to mediate cell adhesion;^24^ ^25^ therefore, we investigated whether RA-SF, OA-SF and exogenous HA influenced adhesion of SF-MSCs. SF samples were pretreated with hyase. The OA-SF hyase treatment markedly increased SF-MSC adhesion, (mean 1.6-fold increase ±0.2 SD, p=0.04, figure 1C). In contrast, hyase treatment of RA-SF had no effect. Comparing hyase treatment of OA-SF and RA-SF highlighted a clear difference (p=0.008, figure 1D) between the adhesion of SF-MSCs in these two types of fluid.

To further clarify the role of HA and to specifically exclude another unanticipated effect of hyase, we used purified, commercially obtained preparations of high and low MW HA (HMWHA and LMWHA, respectively) to supplement culture medium. We observed that, only HMWHA inhibited SF-MSC adhesion from three different donors (figure 1E).

### SF-HA mediates MSC Adhesion to cartilage

**Figure 2:**
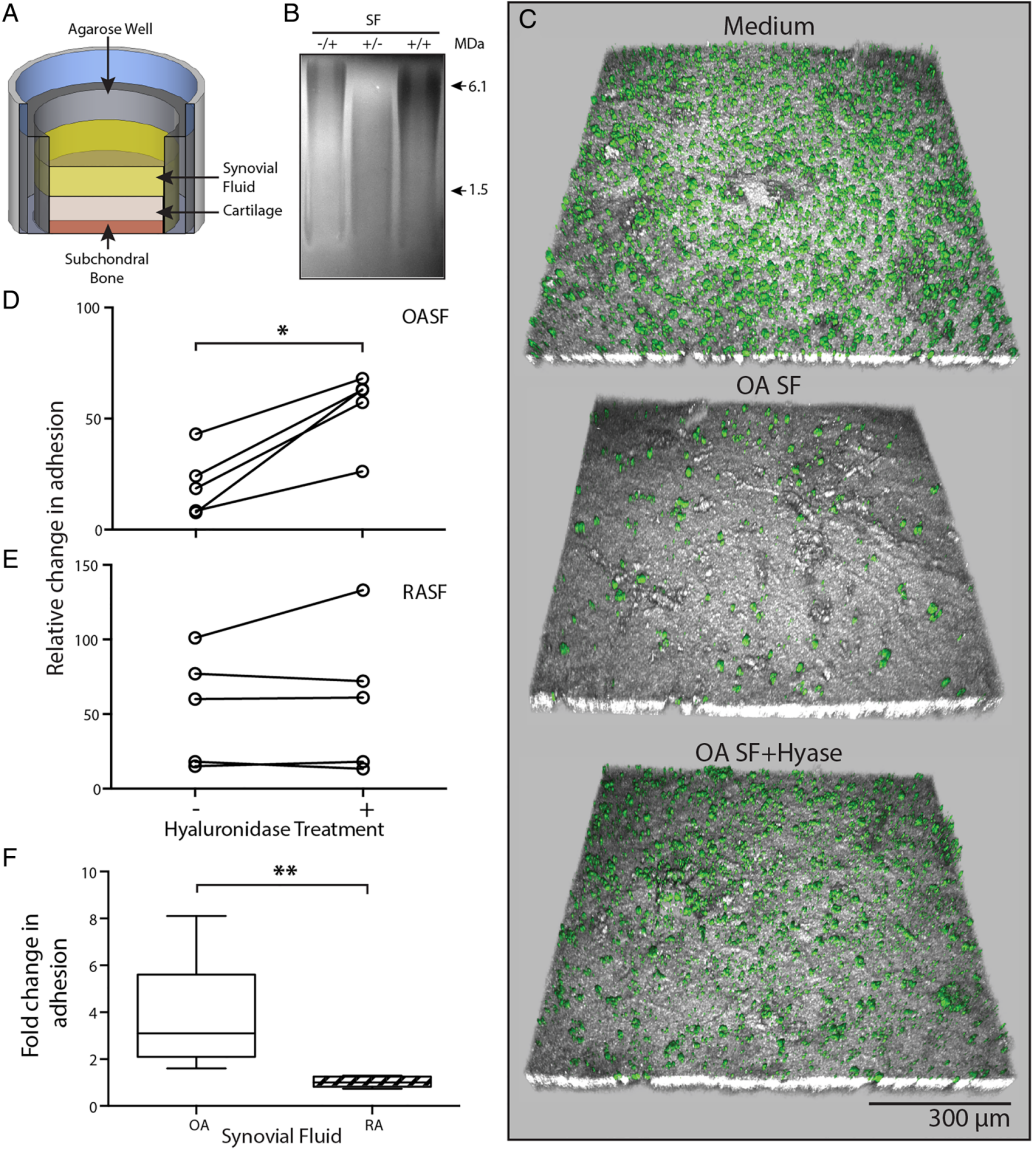
Osteoarthritic (OA) environment limits mesenchymal stromal cell (MSC) attached to cartilage surface. (A) Experimental setup of our novel in vitro adhesion assay. (B) Agarose gel electrophoresis illustrating inhibition of hyase by heparin. Heparin only, −/+; hyase only, +/−; heparin and hyase, +/+. (C) Representative confocal images showing a topographical view of adherent fluorescent micro-sized particles of iron oxide (FMPIO) labelled synovial fluid-mesenchymal stromal cells (SF-MSCs) (green) to superficial cartilage surface (grey), in the presence of culture medium (top), osteoarthritic-synovial fluid (OA-SF) (middle) and after pre-hyase treatment of OA-SF (bottom). (D) Quantification of labelled-MSC adhesion to cartilage surface relative to adhesion in culture medium, showing the consistent increase in adhesion upon pre-hyase digestion of OA-SF (n=5, paired analysis). (E) Equivalent data as in (D) showing SF-MSC adhesion to cartilage in rheumatoid arthritic-synovial fluid (RA-SF) (±hyase pretreatment). (F) Relative differences in adhesion expressed as a fold change of labelled MSCs adhered to cartilage surface after hyase treatment of RA-SF and OA-SF (n=5 each, non-paired analysis). *p<0.05, **p<0.001; individual samples are given in (D) and (E), median values with 25% (box) and 75% (whiskers) CI are presented in (F) and (D).

We used FMPIO labelled-MSCs in a novel in vitro adhesion model with OA derived cartilage, SF and SF-MSCs. Labelled SF-MSCs (see online supplementary figure S1) were added to the joint-facing surface of OC plugs in RA-SF or OA-SF with or without prior digestion of SF with hyase (figure 2A). So as not to digest HA content of the cartilage, heparin, a known inhibitor of hyase was added to all samples prior to the addition of SF-MSC to OC plugs.^28^ The inhibitory effect of heparin is demonstrated in figure 2B compared with non-digested-SF, by the maintenance of HMWHA species (similar to non-digested), which are lost without addition of heparin.

Each in vitro adhesion assay included a positive control whereby SF-MSCs were resuspended and added to OC plugs in culture medium used to normalise adhesion seen in SF and SF+hyase experiments (culture medium samples representing 100% adhesion). Representative confocal images from one adhesion assay using OA-SF are shown in figure 2C. An almost confluent layer of cells can be seen in the positive control image. Upon incubation of SF-MSCs in OA-SF the number of cells adhered is markedly reduced. Adhesion was recovered by pretreating OA-SF with hyase, to levels approaching that of the control. This clear increase in SF-MSC adhesion after SF hyase treatment was consistent across each OA-SF donor (p=0.042, figure 2D), with a mean fold-change increase of 3.7 (±2.3 SD). In contrast, and consistent with experiments on plastic, hyase treatment of RA-SF did not increase SF-MSCs adherence (figure 2E). This clear difference in the effect of hyase between OA-SF and RA-SF (p=0.008, figure 2F) confirms that the antiadhesive nature of OA-SF extends to inhibiting SF-MSC adhesion to cartilage. Increases in adhesion seen in OA-SF were confirmed to be a result of hyase activity rather than toll-like receptor 4 (TLR4) signalling, known to induce adhesion of some cell types^29–31^ (see online supplementary figure S2).

### Osteoarthritic-derived SF induces formation of an HA pericellular coat

**Figure 3:**
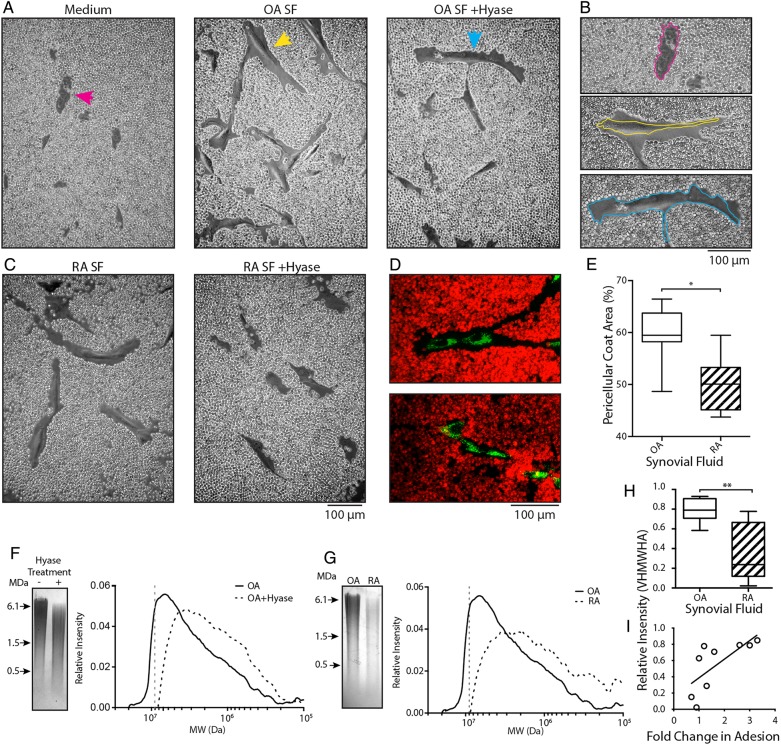
Synovial fluid promotes pericellular hyaluronan-coat formation and is responsible for differences in adhesion to cartilage. (A) Red blood cell (RBC) exclusion assay showing synovial fluid-mesenchymal stromal cells (SF-MSCs) without a hyaluronic acid (HA)-coat in culture medium (left, magenta arrow). SF-MSCs and the presence of an HA-coat after exposure to 10% OA-SF (middle, yellow arrow). Predigestion of OA-SF with hyase fails to induce formation of the HA-coat (right, blue arrow). (B) Highlighted cells from (A), with perimeter of the cell indicated by the coloured line. (C) RBC exclusion assay using rheumatoid arthritic (RA)-derived SF without hyase digestion (left); after prior digestion with hyase (right). (D) Confocal microscopy images of SF-MSCs exposed to osteoarthritic-synovial fluid (OA-SF) (top) and hyase treated OA-SF (bottom) confirming the presence and absence of the HA-coat under these conditions. SF-MSCs are stained green and RBCs stained red. (E) Quantification of the HA-coat area formed by native OA-SF and rheumatoid arthritic-synovial fluid (RA-SF) (n=7 and n=6, respectively; 109 measurements, 40 from OA and 69 from RA donors, non-paired analysis). (F) Representative gel electrophoresis and associated densitometry plot for OA-SF with and without hyase digestion. (G) Representative gel electrophoresis and corresponding densitometry plots showing example OA-SF compared with RA-SF. (H) Relative intensity of very high molecular weight HA (VHMWHA) (>9 MDa HA, represented by the grey dotted line in (F) and (G)) between native OA-SF and RA-SFs used in our in vitro adhesion assay (n=5 each by non-paired analysis). (I) Correlation between proportion of VHMWHA and fold change in adhesion, showing hyase treatment increases adhesion in those SF with more VHMWHA (r=0.88, p=0.002 n=9, non-parametric analysis). *p>0.05, **p<0.005.

The HA-coat is an important mediator in the initial stages of cell adhesion.^24^ ^25^ ^32^ Given the dramatic effect of hyase treatment on OA-SF and SF-MSC adhesion, we determined if exposure of SF-MSCs to SF induced HA-coat formation. Preadhered SF-MSCs were overlaid with culture medium containing 0%, 10%, 20% or 30% OA-SF or RA-SF (±hyase pretreatment, supplemented with heparin). After overnight incubation, these were replaced with fixed red blood cells (RBCs) and allowed to settle under gravity. RBCs formed a clear exclusion zone around MSCs exposed to non-digested (native) OA-SF, which impeded the progression of the RBCs towards the cell (figure 3A, B). These exclusion zones indicate the presence of an HA-coat and are further confirmed following enzymatic digestion with hyase (Movie S1). Culture medium alone or culture medium supplemented with hyase treated OA-SF and RA-SF abolished HA-coat formation (figure 3A–D). SF-MSCs exposed to RA-SF also exhibited an HA-coat; however, these exclusions zones were significantly smaller than that formed in OA-SF. The HA-coat accounted for 59.3%±5.6 and 50.0%±5.5 of the RBC exclusion zones in OA-SF (n=7) and RA-SF (n=6), respectively (means±S.D, figure 3E, p=0.038).

### Increases in MSC adhesion correlate with very high MW HA in SF

Having identified a clear difference between OA-SF and RA-SF on the adhesion and induction of HA-coat formation with SF-MSCs, we further analysed the HA component of SFs. Agarose gel electrophoresis and densitometry were used to investigate the HA content of OA-SF and RA-SF (figure 3F, G), comparing the MW and relative abundance of HA within each SF.^33^ A clear difference in HA content of native and hyase treated SF was seen, with native SF having more abundant, higher MW species (figure 3F). A similar difference was also seen between native non-hyase digested OA-SF and RA-SF (figure 3G), indicating that OA-SF has more abundant HMWHA. Analysis of SF used in our in vitro assay demonstrated a difference in the proportion of very high MW HA (VHMWHA, >9 MDa) between OA-SF and RA-SF (figure 3H, p=0.008).

We next sought to determine if this difference in VHMWHA correlated with the difference seen between changes in MSC adhesion to cartilage upon hyase digestion. A direct correlation between the proportion of VHMWHA in native SF and the fold change in adhesion after digestion of SF with hyase was seen (figure 3I, r=0.88, p=0.002).

### Knee joint distraction modulates SF-HA in vivo in the Canine Groove model

**Figure 4:**
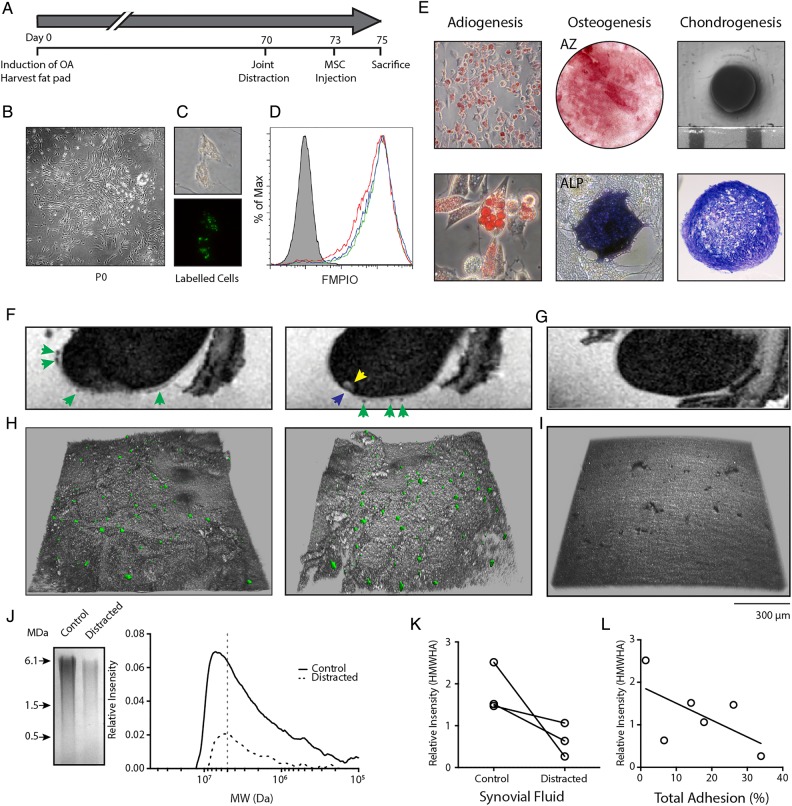
In vivo osteoarthritis model and mesenchymal stromal cell (MSC) adhesion to cartilage under knee joint distraction. (A) Experimental setup of in vivo adhesion model; phases in the experiment are indicated in days. (B) Adipose tissue derived (AT)-MSC colony grown from canine fat pad digest. (C) Fluorescent micro-sized particles of iron oxide (FMPIO)-labelling of AT-MSCs (phase contrast, top; fluorescence, bottom). (D) Flow cytometry analysis of FMPIO uptake by AT-MSCs from each dog (green, red and blue histograms; grey histogram is non-labelled cells from a single dog). (E) Tri-lineage differentiation of AT-MSCs showing adiopgenesis (top and bottom), osteogenesis (AZ, alizarin red; ALP, alkaline phosphatase staining) and chondrogenesis (gross morphology, top; toluidine blue stained section, bottom). (F) Sagittal-MRI of a femur from a distracted limb showing presence of FMPIO-labelled MSCs (green arrows), a bone marrow lesion (yellow arrow) and cartilage defect (blue arrow). (G) Sagittal-MRI of a control joint without the addition of FMPIO-labelled AT-MSCs. (H) Topographical confocal images of cartilage defects adjacent to the bone marrow lesion in (F) showing the presence of FMPIO-labelled MSCs. (I) Topographical confocal image of control cartilage seen in (G). (J) Example gel electrophoresis and associated densitometry plot of synovial fluid (SF) from a matched contralateral control and distracted joint (>7 MDa, grey dotted line). (K) Relative proportion of high molecular weight hyaluronan (HMWHA) (>7 MDa) in each control and distracted joint. Matched animals are indicated by the black line. (L) Correlation between HMWHA in these joints and the percentage of total MSCs adhered per mm^2^ of imaged joint surface. r=−0.6, p=0.2, n=6, non-parametric analysis.

We hypothesised that KJD alters the SF environment in favour of interactions between SF-MSCs and injured cartilage; key stages towards successful colonisation, differentiation and integration, which may contribute to the remarkable cartilage repair seen in humans and animals^9^ ^10^ ^34^ (see online supplementary figure S4). Intra-articular injection of autologous FMPIO-labelled adipose tissue derived (AT) MSCs in the bilateral Canine Groove model was performed and investigated in relation to canine SF-HA MW (figure 4). AT-MSCs used for injection formed colonies (figure 4B) and proliferated in vitro as expected. FMPIO uptake was good, (mean 86%±3.0%, n=3) as determined by flow cytometry (figure 4C, D) and expanded cells displayed tri-lineage differentiation potential (figure 4E).

Osteoarthritic lesions developed on the medial and lateral compartment of the canine femur as well as the untouched tibia after approximately 10 weeks, consistent with previous experience^26^ ^27^ in both the hind limbs of all three dogs. KJD was applied to one hind limb, 3 days prior to intra-articular injection of labelled MSCs. The dogs were sacrificed 48 h after MSC injection (figure 4A).

MRI identified labelled MSCs on the cartilage surface of the femur, as areas of hypointense contrast (dark areas) against the hyperintense contrast (light areas) of the thin cartilage layer (figure 4F) in four of the six joints. No hypointense areas were seen on cartilage surfaces of joints without injection of labelled MSCs (figure 4G). Confocal microscopy was used to investigate the presence of labelled MSCs within each cartilage defect, which was not possible with MRI due to labelled MSCs and areas of denuded bone having similar hypointense contrast and signal intensity (figure 4H). Confocal microscopy revealed MSCs in all six medial compartment defects (distracted and control non-distracted joints), adjacent to bone marrow lesions seen under MRI (yellow arrow, figure 4F).

Gel electrophoresis and densitometry were used to determine the HA content of SF from control and distracted joints (figure 4J). SF-HA was noticeably reduced in all three distracted joints, with HMWHA (>7 MDa) also reduced upon distraction (figure 4K). A trend between the total number of cells detected (using confocal microscopy) and the proportion of HMWHA in SF from control and distracted joints (figure 4L, r=−0.60, p=0.2) was also observed.

## Discussion

Remarkable yet poorly understood repair following KJD has been reported.^9^ ^10^ Herein we demonstrate the potential for SF-MSCs to adhere to cartilage, a prerequisite for SF-MSC mediated colonisation, differentiation and defect repair. Furthermore, we establish the significant role of HA in MSC adhesion in vitro and in vivo, and demonstrate that both inflammation and KJD modulate SF-HA. This knowledge may help explain the success of this emerging treatment, and also offers an insight into how, by providing a window of opportunity we can promote MSC/cartilage interactions towards accelerated cartilage repair. Therefore, as SF has a resident population of MSCs, therapeutic modulation of MSC numbers and their adhesion to cartilage in the OA environment, in isolation or in conjunction with joint biomechanical optimisation might represent a novel cost effective one-stage treatment for OA.

We evaluated SF-HA and MSC adhesion in distracted and non-distracted OA canine joints. Using autologous labelled MSCs, adhesion to cartilage defects in these joints was observed. KJD was accompanied by a decrease in SF-HA in each animal when compared with the control, non-distracted joints, suggesting that increasing the joint space is accompanied by plasma effusion and equilibration of joint pressure, effectively diluting SF-HA. Joint effusion is also seen accompanying KJD in humans throughout the distracted period (personal communication with orthopaedic surgeons P van Roermund and R van Heerwaarde). Consistent with our in vitro data, the abundance of HMWHA species had a negative impact on cell adhesion within these defects. Thus confirming that in vivo HA also influences MSC/cartilage interactions. Finally, in long-term follow-up experiments (without addition of MSCs), KJD showed improved structural outcomes and reduced synovial inflammation indicating restoration of joint function (see online supplementary figure S4),^34^ supporting a recent porcine study also showing MSC adhesion to sites of cartilage injury, cell integration and apparent contribution to subsequent improved histological outcomes.^20^ We cannot definitively conclude that SF-MSCs mediated this repair or discount contribution from cartilage resident or bone marrow MSCs.^35^ However, our data highlights the potential of SF-MSCs to colonise cartilage defects without continued destructive loading under KJD and may be a contributing factor in joint repair.

HA is a major SF constituent, the concentration and MW of which determines SF viscosity. It is known to be reduced in RA and other inflammatory conditions.^36–39^ Using hyaluronidase to specifically digest HA polymers, a significant increase in SF-MSC adhesion was seen in OA-SF only. Comparing adhesion in OA-SF and RA-SF revealed a direct correlation between the highest MW HA species referred to as VHMWHA (>9 MDa), as well as an increase in adhesion after hyase treatment of each SF tested, indicating that VHMWHA in native SF directly influences adhesion of SF-MSCs to cartilage. The relative abundance of MSCs in OA fluid likely reflects these differences by the generally less inflamed environment compared with RA.^16^

The biology of HA has been investigated over several decades with a large body of knowledge having accrued in several fields but its role in MSC adhesion has been neglected. HA interacts with a variety of cell types via CD44 which is highly expressed on SF-MSCs.^15^ ^18^ ^19^ ^21^ ^36^ These interactions form the hyaluronidase sensitive pericellular coat.^22^ ^32^ ^40–42^ More commonly, HA-coats are involved in the initial stages of adhesion,^25^ ^43^ such as extravasation of circulating leucocytes and lymphocytes.^44^ ^45^ Here, low affinity interactions are established via sharing the HA-coat with HA-binding proteins of the substrate.^43^ However, where the substrate also contains a dense HA layer, the presence of an HA-coat inhibits these interactions.^24^ OA-SF induced an HA-coat which was ∼20% larger compared with RA-SF, potentially blocking MSC/cartilage surface interactions.^24^ ^43^ ^46^ ^47^ Thus, the OA environment encourages the formation of a large HA-coat, which may explain why fewer SF-MSCs adhere to cartilage surfaces in our in vitro model, and may also explain why, even with increased SF-MSCs numbers seen in knee injury and OA, joint repair is usually ineffective.^15^ ^16^ ^18^ ^19^

Inflammation and tissue injury is associated with HA breakdown^38^ ^48^ and is further supported here, where increased C reactive protein in RA-SF negatively correlates with VHMWHA abundance (see online supplementary figure S3). LMWHA selectively binds to TLR4, which can stimulate MSC migration and vascular cell adhesion molecule-1 (CD106) expression.^30^ ^49^ Another potent agonist of TLR4 is lipopolysaccharide (LPS), a potential bacterial contaminant of hyase. To rule out LMWHA or LPS involvement in SF-MSC adhesion, we repeated our plastic adhesion assays, supplementing culture medium with HMW and LWMHA and used heat inactivated hyase in this in vitro cartilage model. We also measured by flow cytometry, phenotypic changes to MSCs exposed to ±hyase digested SF. Only HMWHA inhibited SF-MSC adhesion, no effect of heat inactivated hyase on MSC/cartilage adhesion was seen and only minor changes in expression of known cartilage adhesion molecules were measured (see online supplementary figure S3),^47^ further confirming the role of HMWHA in MSC/cartilage adhesion.

To conclude, this work opens up novel possibilities for use of both minimally manipulated endogenous as well as culture expanded allogeneic MSCs, allowing their use in more favourable environments encouraging MSC/cartilage interactions. The recognition that joint resident MSCs may be capable of adhering to cartilage in a HA-dependent manner when placed in the correct mechanical environment also provides a potentially novel explanation for the success of KJD in human OA and encourages the development of cost-effective, one-stage joint saving therapies.

## Supplementary Material

Web supplement

Web video

## References

[1] Houard X, Goldring MB, Berenbaum F Homeostatic mechanisms in articular cartilage and role of inflammation in osteoarthritis. Curr Rheumatol Rep 2013;15:375–10. 10.1007/s11926-013-0375-6 24072604 PMC3989071

[2] Wluka AE, Ding C, Jones G, et al The clinical correlates of articular cartilage defects in symptomatic knee osteoarthritis: a prospective study. Rheumatology (Oxford) 2005;44:1311–16. 10.1093/rheumatology/kei018 16030084

[3] Ding C, Garnero P, Cicuttini F, et al Knee cartilage defects: association with early radiographic osteoarthritis, decreased cartilage volume, increased joint surface area and type II collagen breakdown. Osteoarthritis Cartilage 2005;13:198–205. 10.1016/j.joca.2004.11.007 15727885

[4] Saris DBF, Vanlauwe J, Victor J, et al Treatment of symptomatic cartilage defects of the knee: characterized chondrocyte implantation results in better clinical outcome at 36 months in a randomized trial compared to microfracture. Am J Sports Med 2009;37 (Suppl 1):10S–9S. 10.1177/0363546509350694 19846694

[5] Mastbergen SC, Saris DBF, Lafeber FPJG Functional articular cartilage repair: here, near, or is the best approach not yet clear? Nat Rev Rheumatol 2013;9:277–90. 10.1038/nrrheum.2013.29 23507899

[6] Ferruzzi A, Buda R, Cavallo M, et al Cartilage repair procedures associated with high tibial osteotomy in varus knees: clinical results at 11 years’ follow-up. The Knee 2014;21:445–50. 10.1016/j.knee.2013.11.013 24507767

[7] Koshino T, Wada S, Ara Y, et al Regeneration of degenerated articular cartilage after high tibial valgus osteotomy for medial compartmental osteoarthritis of the knee. Knee 2003;10:229–36. 10.1016/S0968-0160(03)00005-X 12893144

[8] Boegård T, Rudling O, Petersson IF, et al Correlation between radiographically diagnosed osteophytes and magnetic resonance detected cartilage defects in the tibiofemoral joint. Ann Rheum Dis 1998;57:401–7. 10.1136/ard.57.7.401 9797566 PMC1752666

[9] Wiegant K, van Roermund PM, Intema F, et al Sustained clinical and structural benefit after joint distraction in the treatment of severe knee osteoarthritis. Osteoarthr Cartil 2013;21:1660–7. 10.1016/j.joca.2013.08.006 23954704

[10] Intema F, Van Roermund PM, Marijnissen ACA, et al Tissue structure modification in knee osteoarthritis by use of joint distraction: an open 1-year pilot study. Ann Rheum Dis 2011;70:1441–6. 10.1136/ard.2010.142364 21565898 PMC3128325

[11] Mollon B, Kandel R, Chahal J, et al The clinical status of cartilage tissue regeneration in humans. Osteoarthritis Cartilage 2013;21:1824–33. 10.1016/j.joca.2013.08.024 24018339

[12] Roelofs AJ, Rocke JPJ, De Bari C Cell-based approaches to joint surface repair: a research perspective. Osteoarthritis Cartilage 2013;21:892–900. 10.1016/j.joca.2013.04.008 23598176 PMC3694304

[13] Bianco P, Cao X, Frenette PS, et al The meaning, the sense and the significance: translating the science of mesenchymal stem cells into medicine. Nat Med 2013;19:35–42. 10.1038/nm.3028 23296015 PMC3998103

[14] Lee CH, Cook JL, Mendelson A, et al Regeneration of the articular surface of the rabbit synovial joint by cell homing: a proof of concept study. Lancet 2010;376:440–8. 10.1016/S0140-6736(10)60668-X 20692530 PMC4035014

[15] Jones EA, Crawford A, English A, et al Synovial fluid mesenchymal stem cells in health and early osteoarthritis: detection and functional evaluation at the single-cell level. Arthritis Rheum 2008;58:1731–40. 10.1002/art.23485 18512779

[16] Jones EA, English A, Henshaw K, et al Enumeration and phenotypic characterization of synovial fluid multipotential mesenchymal progenitor cells in inflammatory and degenerative arthritis. Arthritis Rheum 2004;50:817–27. 10.1002/art.20203 15022324

[17] Matsukura Y, Muneta T, Tsuji K, et al Mesenchymal stem cells in synovial fluid increase after meniscus injury. Clin Orthop Relat Res 2013;472:1357–64. 10.1007/s11999-013-3418-4 24338094 PMC3971249

[18] Morito T, Hara K, Ju Y-J, et al Synovial fluid-derived mesenchymal stem cells increase after intra-articular ligament injury in humans. Rheumatology (Oxford) 2008;47:1137–43. 10.1093/rheumatology/ken114 18390894

[19] Ojima M, Suzuki S, Yamaga M, et al Human mesenchymal stem cells in synovial fluid increase in the knee with degenerated cartilage and osteoarthritis. J Orthop Res 2012;30:943–9. 10.1002/jor.22029 22147634

[20] Nakamura T, Sekiya I, Muneta T, et al Arthroscopic, histological and MRI analyses of cartilage repair after a minimally invasive method of transplantation of allogeneic synovial mesenchymal stromal cells into cartilage defects in pigs. Cytotherapy 2012;14:327–38. 10.3109/14653249.2011.638912 22309371 PMC3296518

[21] Kurose R, Ichinohe S, Tajima G, et al Characterization of human synovial fluid cells of 26 patients with osteoarthritis knee for cartilage repair therapy. Int J Rheum Dis 2010;13:68–74. 10.1111/j.1756-185X.2009.01456.x 20374387

[22] Heldin P, Pertoft H Synthesis and assembly of the Hyaluronan-containing coats around normal human Mesothelial cells. Exp Cell Res 1993;208:422–9. 10.1006/excr.1993.1264 8375471

[23] Toole BP Hyaluronan: from extracellular glue to pericellular cue. Nat Rev Cancer 2004;4:528–39. 10.1038/nrc1391 15229478

[24] Zimmerman E, Geiger B, Addadi L Initial stages of cell-matrix adhesion can be mediated and modulated by cell-surface hyaluronan. Biophys J 2002;82:1848–57. 10.1016/S0006-3495(02)75535-5 11916844 PMC1301982

[25] Cohen M, Kam Z, Addadi L, et al Dynamic study of the transition from hyaluronan- to integrin-mediated adhesion in chondrocytes. EMBO J 2006;25:302–11. 10.1038/sj.emboj.7600960 16407968 PMC1383524

[26] Mastbergen SC, Marijnissen AC, Vianen ME, et al The canine “groove” model of osteoarthritis is more than simply the expression of surgically applied damage. Osteoarthritis Cartilage 2006;14:39–46. 10.1016/j.joca.2004.07.009 16188467

[27] Intema F, DeGroot J, Elshof B, et al The canine bilateral groove model of osteoarthritis. J Orthop Res 2008;26:1471–7. 10.1002/jor.20681 18473386

[28] Mio K, Stern R Inhibitors of the hyaluronidases. Matrix Biol 2002;21:31–7. 10.1016/S0945-053X(01)00185-8 11827790

[29] Hsu RYC, Chan CHF, Spicer JD, et al LPS-induced TLR4 signaling in human colorectal cancer cells increases beta1 integrin-mediated cell adhesion and liver metastasis. Cancer Res 2011;71:1989–98. 10.1158/0008-5472.CAN-10-2833 21363926

[30] Wu C-Y, Chi P-L, Hsieh H-L, et al TLR4-dependent induction of vascular adhesion molecule-1 in rheumatoid arthritis synovial fibroblasts: Roles of cytosolic phospholipase A(2)alpha/cyclooxygenase-2. J Cell Physiol 2010;223:480–91. 20112284 10.1002/jcp.22059

[31] O'Leary DP, Bhatt L, Woolley JF, et al TLR-4 signalling accelerates colon cancer cell adhesion via NF-κB mediated transcriptional up-regulation of Nox-1. PLoS ONE 2012;7:e44176 10.1371/journal.pone.0044176 23071493 PMC3469572

[32] Clarris BJ, Fraser JR On the pericellular zone of some mammalian cells in vitro. Exp Cell Res 1968;49:181–93. 10.1016/0014-4827(68)90530-2 5640690

[33] Cowman MK, Chen CC, Pandya M, et al Improved agarose gel electrophoresis method and molecular mass calculation for high molecular mass hyaluronan. Anal Biochem 2011;417:50–6. 10.1016/j.ab.2011.05.023 21683677

[34] Wiegant K, Intema F, Van Roermund PM, et al Evidence for cartilage repair by joint distraction in a canine model of osteoarthritis. Arthritis Rheumatol 2015;67:465–74. 10.1002/art.38906 25303046

[35] Williams R, Khan IM, Richardson K, et al Identification and clonal characterisation of a progenitor cell sub-population in normal human articular cartilage. PLoS ONE 2010;5:e13246 10.1371/journal.pone.0013246 20976230 PMC2954799

[36] Necas J, Bartosikova L, Brauner P, et al Hyaluronic acid (hyaluronan): a review. Veterinarni Medicina 2008;53:397–411.

[37] Balazs EA, Watson D, Duff IF, et al Hyaluronic acid in synovial fluid. I. Molecular parameters of hyaluronic acid in normal and arthritic human fluids. Arthritis Rheum 1967;10:357–76. 10.1002/art.1780100407 6046018

[38] Praest BM, Greiling H, Kock R Assay of synovial fluid parameters: hyaluronan concentration as a potential marker for joint diseases. Clin Chim Acta 1997;266:117–28. 10.1016/S0009-8981(97)00122-8 9437540

[39] Saari H, Konttinen YT Determination of synovial fluid hyaluronate concentration and polymerisation by high performance liquid chromatography. Ann Rheum Dis 1989;48:565–70. 10.1136/ard.48.7.565 2774697 PMC1003817

[40] Knudson W, Bartnik E, Knudson CB Assembly of pericellular matrices by COS-7 cells transfected with CD44 lymphocyte-homing receptor genes. Proc Natl Acad Sci USA 1993;90:4003–7. 10.1073/pnas.90.9.4003 8483916 PMC46434

[41] Rilla K, Tiihonen R, Kultti A, et al Pericellular hyaluronan coat visualized in live cells with a fluorescent probe is scaffolded by plasma membrane protrusions. J Histochem Cytochem 2008;56:901–10. 10.1369/jhc.2008.951665 18574248 PMC2544615

[42] Lee GM, Johnstone B, Jacobson K, et al The dynamic structure of the pericellular matrix on living cells. J Cell Biol 1993;123:1899–907. 10.1083/jcb.123.6.1899 8276905 PMC2290877

[43] Cohen M, Joester D, Geiger B, et al Spatial and temporal sequence of events in cell adhesion: from molecular recognition to focal adhesion assembly. Chembiochem 2004;5:1393–9. 10.1002/cbic.200400162 15457530

[44] DeGrendele HC, Estess P, Picker LJ, et al CD44 and its ligand hyaluronate mediate rolling under physiologic flow: a novel lymphocyte-endothelial cell primary adhesion pathway. J Exp Med 1996;183:1119–30. 10.1084/jem.183.3.1119 8642254 PMC2192320

[45] DeGrendele HC, Estess P, Siegelman MH Requirement for CD44 in activated T cell extravasation into an inflammatory site. Science 1997;278:672–5. 10.1126/science.278.5338.672 9381175

[46] Shimaya M, Muneta T, Ichinose S, et al Magnesium enhances adherence and cartilage formation of synovial mesenchymal stem cells through integrins. Osteoarthr Cartil 2010;18:1300–9. 10.1016/j.joca.2010.06.005 20633668

[47] Koga H, Shimaya M, Muneta T, et al Local adherent technique for transplanting mesenchymal stem cells as a potential treatment of cartilage defect. Arthritis Res Ther 2008;10:R84 10.1186/ar2460 18664254 PMC2575632

[48] Jiang DH, Liang JR, Fan J, et al Regulation of lung injury and repair by Toll-like receptors and hyaluronan. Nat Med 2005;11:1173–9. 10.1038/nm1315 16244651

[49] Tomchuck SL, Zwezdaryk KJ, Coffelt SB, et al Toll-Like Receptors on Human Mesenchymal Stem Cells Drive Their Migration and Immunomodulating Responses. Stem Cells 2008;26:99–107. 10.1634/stemcells.2007-0563 17916800 PMC2757778

