# Supplementary material for: Synovial fluid hyaluronan mediates MSC attachment to cartilage, a potential novel mechanism contributing to cartilage repair in osteoarthritis using knee joint distraction
Source: Ann Rheum Dis. 2015 May 6;75(5):908–15. doi: 10.1136/annrheumdis-2014-206847 (PMC4853581; doi:10.1136/annrheumdis-2014-206847)
Supplement: Web supplement [file annrheumdis-2014-206847-s1.pdf]

Synovial fluid hyaluronan mediates MSC attachment to cartilage, a potential novel mechanism contributing to cartilage repair in osteoarthritis using knee joint distraction

Thomas G. Baboolal, Simon C. Mastbergen, Elena Jones, Stuart J. Calder, Floris P.J.G. Lafeber, Dennis McGonagle.

SUPPLEMENTARY METHODS

Collection of human cartilage, SF and SF derived MSC expansion

All samples were collected following informed written consent with relevant ethical approval. Cartilage samples were obtained after total knee replacement surgery for OA. To minimise inter sample variation, cartilage plugs were harvested from macroscopically normal areas of the femoral condyles (usually the lateral compartment) using an 8 mm diameter biopsy punch together with the underlying subchondral bone and washed in phosphate buffered saline (PBS, Invitrogen, Paisley, UK). These osteochondral (OC) plugs and placed in culture medium (DMEM, 10% FCS, 100U penicillin and 100 µg streptomycin) until use with regular changes of medium, for no longer that 1 week.

All SF was either collected from patients undergoing joint replacement surgery or during routine clinics. For SF sampling, all cells and debris were pelleted at 16,000 rcf for five minutes This cell free SF was frozen in aliquots at -80°C until use. For SF derived MSC (SF-MSC) expansion, SF was diluted 1:5 in PBS and cells pelleted at 600 rcf for five minutes before resuspending and seeding (five cm^2 per ml of undiluted SF) in non-haemopoetic expansion (NH) media (Miltenyi Biotec). Cells were passaged using trypsin for five minutes at 37°C and reseeded at a density of 10^4 cells/cm^2 and expanded up to passage 4 (p4) at 37°C in humidified 5% CO2 with twice weekly media changes.

Plastic Adhesion Assay

Cell free SF from OA and RA patients was thawed in a 37°C water bath and centrifuged at 16,000 rcf to remove any residual debris. SF was then either treated with 5 U/mL hyaluronidase (hyase, Sigma-Aldrich) or equal volume of PBS as a control for 30 minutes in a 37°C water bath. After incubation, 10 U/mL lithium heparin (MP Biomedicals, Cambridge, UK) was added to each sample. These were then used to resuspend SF-MSCs (p2-4), and 10^4 cells were added to wells of a 96 well plate in triplicate. Cells seeded in culture medium were used as a positive control. Additionally, culture medium was supplemented with 5 mg/ml of high or low molecular weight hyaluronan (HA2M, 2.6MDa and HA60K, 7.5KDa; high and low molecular weight hyalyronan respectively, LifeCore Biomedical, Minnesota, USA) and serial dilutions where made down to 0.75 mg/ml before addition of SF-MSCs. SF-MSCs were left to adhere overnight before removal of SF or medium and replacing with XTT reagents (according to manufactures instructions, Roche Diagnostics Ltd, Burgess Hill, UK). Cells were incubated with XTT reagents at 37°C humidified incubator with 5% CO_2 for four-five hours before absorbance measurements at 490nm and 650nm.

Cell Labelling with Fluorescent Micro-sized Particles of Iron Oxide

We used dual fluorescent (allowing laser confocal imaging) and paramagnetic (for magnetic resonance imaging, MRI) particles for multimodal imaging of MSCs. Passage two to three MSCs derived from canine fat pads (se below) or human knee SF were seeded in six-well plates (approx. 10^5 cells per well) and left to adhere. Fluorescent micro-sized particles of iron oxide (FMPIO, Bang Laboragories, Indiana, USA) were prepared by washing in one mL of PBS with removal of supernatant after magnetic immobilization of FMPIO. This was repeated three times with the final resuspension in

culture medium. FMPIO were added at a density of 10^7 per cm^2 and left to internalize overnight. Before use, tagged cells were washed three times with five mL PBS to remove free FMPIO and trypsinised as above. Labeled-MSCs for *in vivo* studies were frozen (90% fetal calf serum, 10% dimethyl sulfoxide) and stored at -80°C until use. SF-MSCs labeled for *In vitro* studies were used immediately.

Labeled SF-MSCs Proliferation Assay

FMPIO have previously been shown to be well tolerated by MSCs [1]. Human SF-MSCs from two different donors (p1 and p2) were labeled with FMPIO as above with increasing numbers of FMPIO per cell (200, 600 and 1000). After overnight incubation, the cells were washed, trypsinised and seeded in 96 well plates at 50, 250 and 1000 cells per well in quadruplet. As a control, cells without FMPIO were seeded at the same densities. All cells were left to proliferate for 8 days at 37°C in humidified 5% CO_2. After which, XTT reagents were added to each well according to manufactures instructions and incubated for four-five hours before absorbance measurements at 490nm and 650nm.

Flow Cytometry of FMPIO Labeled MSCs

Flow cytometry was performed on labeled cells using a FACScan (BD Biosciences, Oxford, UK). SF-MSCs (p2-4) from five different donors were labeled as above. Immediately prior to acquisition dead cells were labeled using 7AAD (BD Biosciences). Approximately 10,000 live cell events were acquired per sample.

Heparin Inhibition of Hyaluronidase

To demonstrate heparin inhibition of hyase, OA-SF was incubated with hyase (5 U/mL) or the equivalent volume of PBS (as above). Additionally hyase treated samples were also treated with the addition of 10 U/mL lithium heparin. Each sample was incubated

overnight in a 37°C water bath. Samples were processed and electrophoresed as described below.

OA Joint Environment Novel Cartilage Adhesion Assay

Macroscopically normal cartilage plugs (8 mm diameter) were prepared as above. Each plug was placed into a preformed 8 mm diameter well of sterile 2% agarose so that the surface of the cartilage was contained within the walls of the well. This was to facilitate SF-MSCs-cartilage surface interaction and to prevent MSC loss down the sides of cartilage plugs. Additionally, the volume of SF containing SF-MSCs was limited to 150µL so that it did not breach the confines of the agarose well. FMPIO-labeled SF-MSCs (p2-4, $5x10^4$ per OC plug) were prepared as above and resuspended in either culture medium, OA- or RA-SF that were treated with or without hyase (see above). Each cartilage plug was given a final wash with PBS before addition of SF and SF-MSCs or culture medium and SF-MSCs, and were incubated overnight at 37°C in a humidified incubator with 5% CO_2. Intra-assay variability was controlled by using the same three donors for cartilage, SF and SF-MSCs using adhesion of MSCs in culture medium as a positive control for each assay. Inter-assay, SF from a different donor was used each time. After an overnight incubation, the SF or culture medium was removed and the cartilage surface gently washed with three exchanges of 0.5 mL PBS. Adherent SF-MSC were fixed to the cartilage surface with overnight incubation in 3.7% formalin and stored at 4°C in PBS supplemented with 3mM sodium azide until use.

Confocal Microscopy of Adherent Labeled-MSCs on Cartilage Surface.

Confocal microscopy using a Leica SP2 TCS laser scanning microscope (Leica, Buckinghamshire, UK) was used to image the cartilage surface topography with adherent FMPIO-labeled SF-MSCs. MSCs were visualized by way of their uptake of

FMPIO, which have an excitation and emission wavelength of 480 and 520 nm respectively. The cartilage surface was visualised using its reflected light characteristics (excitation at 488nm and emission between 479-498nm). Images were collected of hydrated sample (in PBS) using water dipping 10x objective, z-stakes collected with 3 μm optical sections.

Semi-Quantitative Analysis of FMPIO-Labeled MSCs

Image processing of labeled-MSCs was performed using Volocity software version 6.1.1. (Perkin Elmer, Cambridge, UK). Detection of FMPIO-labelled MSCs was based on fluorescent intensity thresholding and size exclusion criteria defined on images of FMPIO-tagged MSCs adhered to untreated glass slides. For detection of nucleus to aid cell counting, nuclear staining of SF-MSCs was performed using 1 μM To-Pro-3 (Invitrogen).

Synovial Fluid Gel Electrophoresis and Densitometry of Hyaluronan

Agarose gel electrophoresis was performed according to the methods of Cowman et al (2011). Briefly, 0.5% agarose gel was cast using Tris acetate EDTA buffer (TAE, 40mM Tris, 5mM acetate and 0.9mM EDTA, pH7.9). SF samples were treated with or without hyase as described above. After which heparin was added to prevent further hyase activity and were incubated overnight at 37°C to simulate conditions of our *in vitro* adhesion assay. To remove the protein content of the SF, each sample was digest with an equal volume of Pronase (1500 PKU/ml, Roche Diagnostics Ltd) for five hours at 37°C the following day. Samples were stored at -20°C until use. Each sample of SF (3 μL) was mixed with water (10 μL) and loading dye (3 μL, 0.02% bromophenol blue, 2M sucrose, in TAE), to ensure equal loading between samples 10 μL of the sample mixture was loaded per well. For molecular weight determination, 5 μL of each Select-HA

ladders (MegaLadder, HiLadder and LowLadder, Sigma-Aldrich) and 3 μL loading dye were loaded per gel.

Electrophoresis was performed in TAE buffer, initially for 30 mins at 20 V and then for an additional 3.5 hours at 40 V. Immediately after, the gel was placed in 0.005% Stains-All (in 50% ethanol) at room temperature overnight and protected from the light. Destaining was performed in 10% ethanol for 9 hrs in the dark with at least one change of destaining solution [2]. The gel was then exposed to light to remove residual background and digitized with using GelDoc XR and Quantity One software (version 4.6.9 BioRad Life Sciences, Hemel Hempstead, UK).

Quantitative analysis was again performed using the method of Cowman et al (2011). Average intensity profiles across the width of each well for each sample were generated using ImageJ 64 (version 1.43u) [3] and background corrected. A calibration plot was created using the logarithm of the molecular weight for each Select-HA marker and their migration distance in pixels. This was used to convert migration distance (in pixels) for each sample into molecular weight. To compare relative intensities in canine SF, each sample was normalized to the total area for all samples on the sample gel. Human SF sample were individually normilised to the area under the curve for each sample [2].

Flow Cytometery Analysis of SF-MSCs Exposed to Hyaluronidase Treated SF

SF-MSCs (p4) from a single donor were pre-adhered to six well plates. SFs from different donors were treated with 5 U/mL hyase or equivalent volume PBS (RA n=3, OA n=3) for 30 min at 37°C as previously described. A 20% solution of each SF (±hyase) was made using culture medium supplemented with 10 U/mL heparin. Cells were incubated with these solutions as well as culture medium (±hyase and heparin) overnight at 37°C, 5% CO_2. The following day, SF/medium was removed, cells washed in PBS,

trysonised and resuspended in FACS buffer (PBS, 0.5% bovine serum albumin (BSA) and 2 mM EDTA). Cells were stained with the following antibodies; CD44-phycoerythrin (PE), CD54-PE, CD106-PE and CD166-PE and appropriate isotype controls (all BD Biosciences, Oxford, UK). Cells were stained with DAPI (Sigma-Aldrich, Dorset, UK) as a live/dead discriminator immediately prior to acquisition. Data were collected using LSRII and analysed using FACSDiva software version 7.

Red Blood Cell Exclusion Assay and Pericellular HA-coat Quantification

Pericellular HA-coat formation in the presence of SF was visualized using pre-adhered SF-MSCs (p2) at a density of $2x10^4$ cell per well in 24 well tissue culture plate. To each well OA-SF was added (10, 20 and 30% v/v in culture medium and 10 U/ml heparin) with or without prior digestion with hyase (as above). As a control, culture medium and culture medium supplemented with heparin and hyase was also added. The cells were incubated overnight to allow HA-coat formation. HA-coat formation was detected as previously described [4]. Briefly, human red blood cells (RBCs) were fixed overnight in 2% formalin before washed and resuspended in PBS containing 0.1% BSA. Medium/SF mixes were carefully removed from each well and fix RBCs were added at a density of $5x10^7$ RBCs per well and left to settle under gravity. The cells were visualized using an inverted microscope. To further confirm the presence of the HA-coat SF-MSCs and RBCs were labeled with the fluorescent membrane dyes PKH67 and PKH26 respectively, according to manufacturers instructions (Sigma-Aldrich). Cells treated with OA-SF with or with hyase treatment as above and imaged using a Nikon A1R laser canning confocal microscope. Time-lapse movie showing the degradation of the HA-coat was created using iMovie (Version 10.0.6, Apple inc, USA) from still images taken at 10 minute intervals after the addition of hyase (5 U/ml) to pre-adhered SF-MSCs.

Coat area was measure from digitized images. Indirect measurements were made by determining the area of a cell and subtracting that from the total area of the RBC exclusion zone (which included the cell, using ImageJ 64 (version 1.43u) [3]. A total of 40 images, containing 137 RBC exclusion zones were analysed from 13 different SF samples (n=7 OA and n=6 RA-SF).

Synovial Fluid CRP Measurements

C-reactive protein (CRP) levels of SF were measured to assess the inflammatory status of the patient. Measurements made included those SF used in our *in vitro* model. To aid dilution of the SF, each SF was digested with hyase (5 U/mL) overnight at 37°C. This aided mixing of the highly viscid fluid by reducing viscosity. Measurements were made using a Quantikine ELISA for the detection of human CRP according to manufacturers instructions (R&D Systems, Oxfordshire, UK).

Animals

Skeletally mature mixed breed dogs (all females and skeletally mature) were obtained from the animal laboratory of Utrecht University, the Netherlands. Dogs were housed together (2-3 per 12 m^2), and were exercised in groups (in an area of 48 m^2) daily for at least two hours. Dogs were fed on a standard diet with water *ad libitum*. The Utrecht University Committee of Experiments on Animals approved the study according to Dutch law (DEC: 2011.III.11.116).

Isolation and Culture Expansion of Adipose Tissue Derived MSCs from Canine Groove Model of OA

Adipose tissue was removed from the canine fat pad at the time of initial surgical procedure for OA induction. Fat pad were and sent to the UK on ice. Within 48 hours

each fat pad was minced and digested with equal v:w collagenase (Stemcell Technologies, Grenoble, France) at 37°C for four hours with constant agitation to obtain a single cell suspension. Undigested tissue was removed using a 70 μm cell strainer (BD Biosciences). Cells were washed in 10 ml medium (DMEM, 10% FCS, pen/strep) and pelleted at 600 x rcf for 10 minutes. Pelleted cells were cultured for eight-14 days in culture medium before counting colonies. For passaging, cells were detached using trypsin for five minutes at 37°C and reseeded at a density of $1.5x10^5$ cells/cm^2 and expanded up to passage 3 (p3) at 37°C in humidified 5% CO2 with twice weekly media changes.

Trilineage Differentiation of Canine Adipose Derived MSCs

Differentiation assay were performed on p2 cells. For chondrogenesis assay, $2.5x10^5$ cells were pelleted by centrifugation before carefully overlaying the pellet with chondrogenic medium (100 μg/ml sodium pyruvate, 40 μg/ml proline, 50 μg/ml L-ascorbic acid-2- phosphate, 1 mg/ml BSA, 1x insulin–transferrin–selenium plus, 100 n*M* dexamethasone (all from Sigma-Aldrich), and 10 ng/ml transforming growth factor β3 (TGFβ3; R&D Systems). Half media changes were performed three times a week and micromasses were harvested after 21 days. For histology, 5 μm frozen sections were stained with 1% toluidine blue (Sigma-Aldrich) to stain for glycosaminoglycan content [5].

For osteogenic and adipogenic differentiation, cells were plated in six-well plates at a density of 10^3 cells/cm^2 and cultured for 21 days in either osteogenic (DMEM (Invitrogen), 10% FCS (Biosera, Uckfield, UK), 100 nM dexamethasone, 0.05 mM L-ascorbic acid-2-phosphate, and 10 mM β-glycerophosphate (all from Sigma-Alrdich) or adipogenic differentiation medium DMEM (Invitrogen), 10% FCS, 10% horse serum (Stem Cell Tech), 60 μM indomethacin (ICN, Basingstoke, UK), 0.5 mM

isobutylmethylxanthine, and 0.5 mM hydrocortisone (Sigma-Aldrich). Commitment towards the osteogenic lineage was shown by staining for alkaline phosphatase activity (after 14 days) using the Sigma kit 82 (Sigma-Aldrich) and matrix mineralisation with 1% alizarin red [6]. Accumulation of lipid vesicles was visualised using oil red [7,8].

Knee Joint Distraction in Canine Groove Model and Injection of Labelled MSCs

In all three dogs, OA was bilaterally induced in the right and left stifle joint according to the canine Groove model [9,10]. After ten weeks in which joint degeneration developed also on the untouched tibial plateaus, knee KJD was performed on the right stifle joint for five days by use of an external fixation frame. The external fixation frame was placed under general anesthesia and pain medication. The fixation frames of femur and tibia were connected by hinges medially and laterally of the knee joint. By use of screw-threaded connecting rods, distraction of the knee joint was carried out and visualized by fluoroscopy using a C-arm, while smooth motion of the joint during flexion and extension was maintained.

Frozen, FMPIO-labelled MSCs (p2) were recovered into DMEM supplemented with 10% canine autologous derived serum. Cells were pelleted at 600 x rfc for five minutes and resuspended in saline supplemented with 5% canine serum. After washing cells were loaded into a syringe in a final volume 1 ml. Each knee revised between 5.6-8.7x10^6 autologous cells injected into the synovial cavity between the articulating surfaces of both legs of each animal 72 hours after placement of the external fixation frames. Each animal was allowed to continue normal daily activities before euthanizing 48 hours after MSC injection whereby the joints were dissected and fixed in formalin avoiding contact with the articular surface.

MRI of Canine Joints

MRI of the distal femur was performed using a 3T Siemens Verio scanner (Siemens Healthcare, Erlangen, Germany). The dual echo steady-state (DESS) gradient echo image sequence employed a 140 x 140mm field of view, an imaging matrix of 384 x 384 px and a slice thickness of 370 μm. T2-wighted images were acquired using an echo time (T_E) of 4.7 msec, repetition time (T_R) of 16.3 msec and a flip angle of 25°. Images where collected of the six femoral heads and areas of hypointenisty due to the presence of labelled cels were identified using OsiriX DICOM Viewer, version 4.0 [11].

Long Term Follow up of Knee Joint Distraction in Canine Groove Model

Induction of OA was according to the canine Groove model [9,12]. Animals were placed into two groups; control with OA induction but without KJD (n=9) and KJD group (n=9) as described above, KJD was applied after 10 weeks of development of OA and performed for 10 weeks without the addition of AT-MACs. After removal of the frame an additional follow-up of 25 weeks was allowed and animals were euthanized at week 45. Macroscopic cartilage damage and synovial tissue inflammation were graded according to the OARSI score [13]. Two independent observers performed macroscopic evaluations on blinded high-resolution digital photographs of cartilage and synovium from OA and KJD groups.

Statistical Analysis

All statistics were performed using SPSS Statistics (IBM, Portsmouth, Version 21). Unless otherwise stated, due to the low sample numbers, all data was treated as non-parametric. Where applicable, paired analysis was done using the Wilcoxon Signed Ranks and non-paired using the Mann-Whitney U test. Correlations were done using the Spearman's rank correlation coefficient for non-parametric data

SUPPLEMENTARY DATA

Figure S1: FMPIO-Labelling and Detection of SF-MSCs. A) Example confocal image showing FMPIO-labelled SF-MSCs. FMPIO in green, MSC nucleus stained with To-Pro3, in blue. B) Cell proliferation assay showing no cytotoxic affects of labelling SF-MSCs with increasing FMPIO concentration per cell. C) Flow-cytometry showing labelling efficiency for a representative donor. Labelling was high with an average of 94.3% (± 2.3%, n=5) having uptake of FMPIO. D) Confocal microscopy of labelled-MSCs showing detection of nucleus (red) and FMPIO (purple) using Volocity software. E) Quantification and correlation of FMPIO volume and cell number (p=<0.0001, r=0.96, n=1376 cells). F) Example confocal image showing detection of FMPIO-labelled SF-MSCs attached to cartilage. Analysis protocol detects labelled SF-MSCs based on size and fluorescence intensity ensuring no false detection of the cartilage surface.

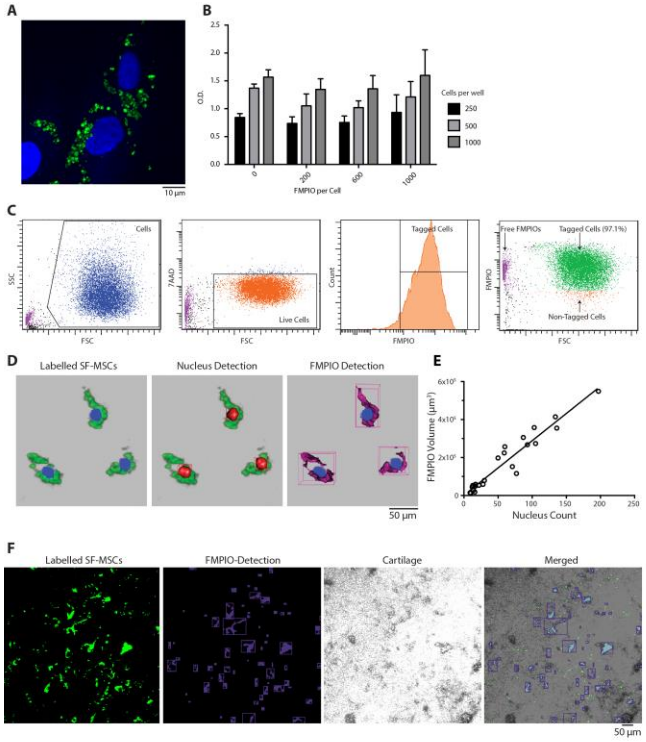

Figure S2: Confirmation that Adhesion Changes are due to Digestion of VHMWHA. A) Gel electrophoresis of OA-SF digested with active and heat denature hyase. B) Associated densitometry plot from (A). C) Quantification of SF-MSC adhesion to cartilage showing an increase in adhesion due to active hyase enzyme. D) Example topographical confocal images of the cartilage surface from the data presented in (C). E) Flow cytometry analysis comparing the mean fluorescence intensity (MFI) of cartilage adhesion proteins from a single SF-MSC donor with different OA (n=3) and RA (n=3) SF donors with and without hyase treatment together with SF-MSCs exposed to culture medium (CM) with and without hyase and heparin (CM++). F) Flow cytometry analysis comparing percentage of cell expressing cartilage adhesion proteins in OA-, RA-SF and culture medium as in (E).

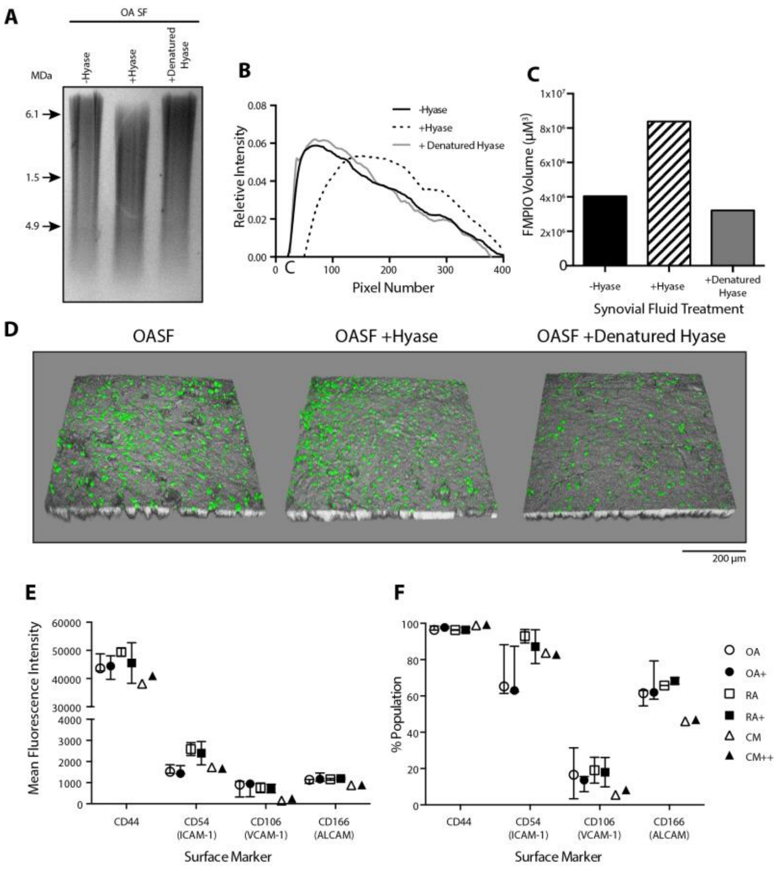

Figure S3: Inflammation is Associated with Synovial Fluid Hyaluronan Content, A) CRP levels measured in OA (n=7) and RA SF (n=6). B) Correlation between SF CRP and VHMWHA (r=-0.6, p=0.036, n=12). C) Relationship between SF CRP and MSC adhesion for the SF use in the *in vitro* cartilage adhesion model (n=9).

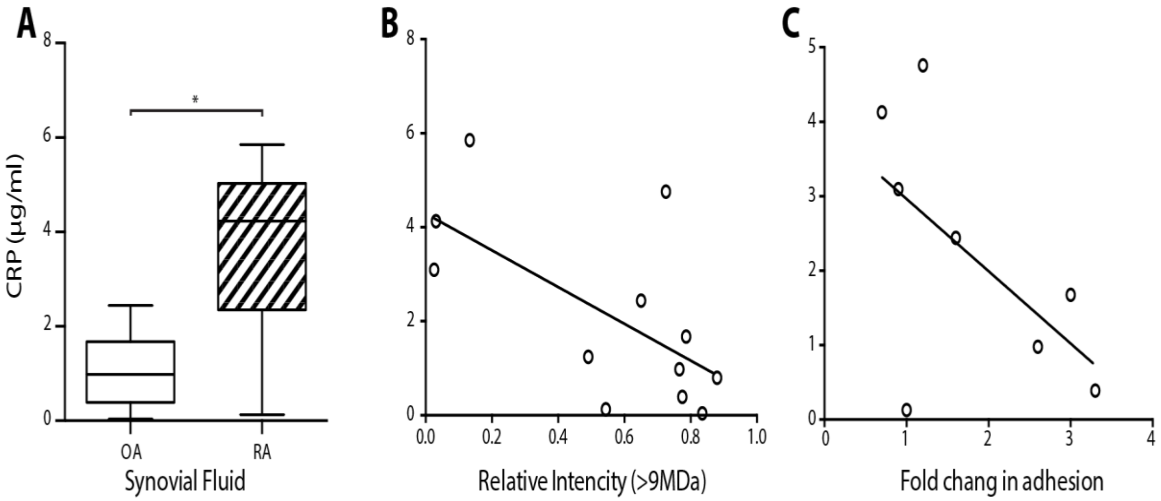

Figure S4: Knee Joint Distraction is Associated with Improvements in Cartilage Integrity and Synovial Inflammation. A) Experimental setup for our *in vivo* cartilage repair model, phases in the experiment are indicated in weeks. B) Example of images of cartilage integrity and synovial inflammation seen in the *in vivo* model after 45 weeks. Experimentally induced OA (10 weeks) without (left) and with KJD (10 weeks, right) at subsequent 25 weeks of follow-up C) Macroscopic cartilage damage and synovial tissue inflammation were graded according to the OARSI score (*p<0.05, n=9, non-paired parametric analysis) [13].

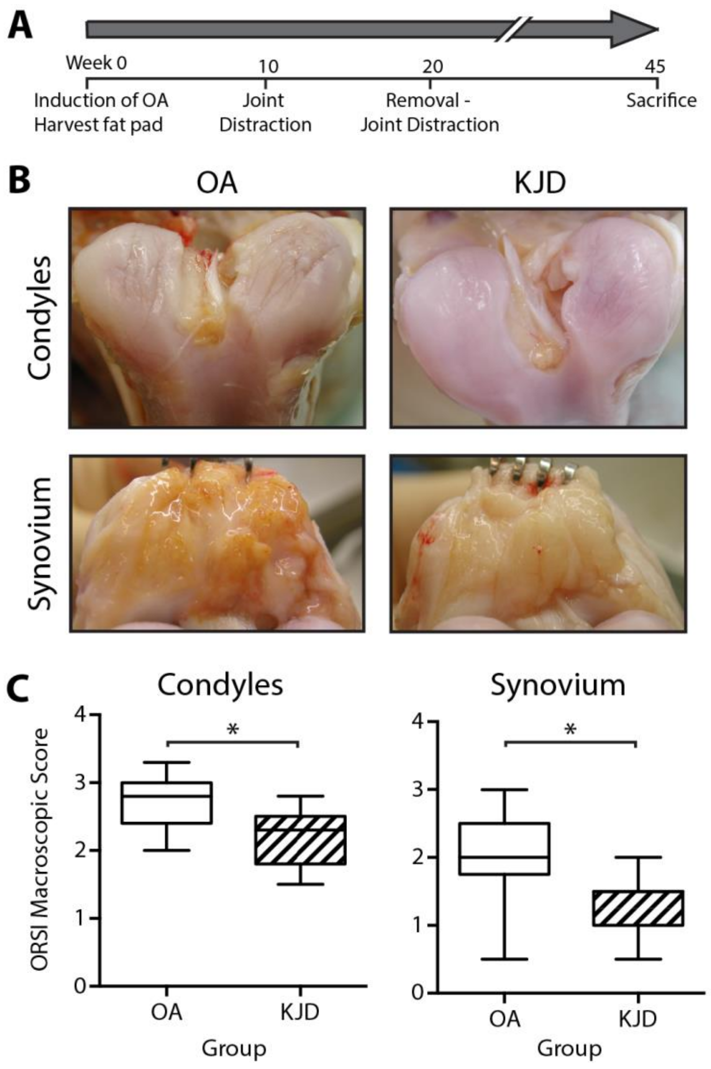

Supplementary Movie S1: Degradation of the Hyaluronan Pericellular Coat. Time-lapse movie showing how the RBC exclusion zone is abolished over time (up to 70 minutes) after the addition of hyaluronidase, which digests the HA within the pericellular coat surrounding each MSC.

REFERENCES

1 Boulland J-L, Leung DSY, Thuen M, *et al.* Evaluation of intracellular labeling with micron-sized particles of iron oxide (MPIOs) as a general tool for in vitro and in vivo tracking of human stem and progenitor cells. *Cell Transplant* 2012;**21**:1743–59. doi:10.3727/096368911X627598

2 Cowman MK, Chen CC, Pandya M, *et al.* Improved agarose gel electrophoresis method and molecular mass calculation for high molecular mass hyaluronan. *Anal Biochem* 2011;**417**:50–6. doi:10.1016/j.ab.2011.05.023

3 Schneider CA, Rasband WS, Eliceiri KW. NIH Image to ImageJ: 25 years of image analysis. *Nat Methods* 2012;**9**:671–5.

4 Heldin P, Pertoft H. Synthesis and Assembly of the Hyaluronan-Containing Coats Around Normal Human Mesothelial Cells. *Exp Cell Res* 1993;**208**:422–9. doi:10.1006/excr.1993.1264

5 Jones EA, English A, Henshaw K, *et al.* Enumeration and phenotypic characterization of synovial fluid multipotential mesenchymal progenitor cells in inflammatory and degenerative arthritis. *Arthritis Rheum* 2004;**50**:817–27. doi:10.1002/art.20203

6 Jaiswal N, Haynesworth SE, Caplan AI, *et al.* Osteogenic differentiation of purified, culture expanded human mesenchymal stem cells in vitro. *J Cell Biochem* 1997;**64**:295–312. doi:10.1002/(SICI)1097-4644(199702)64:2<295::AID-JCB12>3.0.CO;2-I

7 Pittenger MF, Mackay AM, Beck SC, *et al.* Multilineage potential of adult human mesenchymal stem cells. *Science* 1999;**284**:143–7.

8 Ramírez-Zacarías JL, Castro-Muñozledo F, Kuri-Harcuch W. Quantitation of adipose conversion and triglycerides by staining intracytoplasmic lipids with oil red O. *Histochemistry* 1992;**97**:493–7. doi:10.1007/BF00316069

9 Mastbergen SC, Marijnissen AC, Vianen ME, *et al.* The canine “groove” model of osteoarthritis is more than simply the expression of surgically applied damage. *Osteoarthritis and Cartilage* 2006;**14**:39–46. doi:10.1016/j.joca.2004.07.009

10 Intema F, DeGroot J, Elshof B, *et al.* The canine bilateral groove model of osteoarthritis. *J Orthop Res* 2008;**26**:1471–7. doi:10.1002/jor.20681

11 Rosset A, Spadola L, Ratib O. OsiriX: An Open-Source Software for Navigating in Multidimensional DICOM Images. *J Digit Imaging* 2004;**17**:205–16. doi:10.1007/s10278-004-1014-6

12 Wiegant K, Intema F, Van Roermund PM, *et al.* Evidence for cartilage repair by joint distraction in a canine model of osteoarthritis. *Arthritis Rheumatol* 2014;:n/a–n/a. doi:10.1002/art.38906

13 Cook JL, Kuroki K, Visco D, *et al.* The OARSI histopathology initiative - recommendations for histological assessments of osteoarthritis in the dog. 2010. S66–79. doi:10.1016/j.joca.2010.04.017
